# Nutritional profile of rodent diets impacts experimental reproducibility in microbiome preclinical research

**DOI:** 10.1038/s41598-020-74460-8

**Published:** 2020-10-20

**Authors:** C. J. Tuck, G. De Palma, K. Takami, B. Brant, A. Caminero, D. E. Reed, J. G. Muir, P. R. Gibson, A. Winterborn, E. F. Verdu, P. Bercik, S. Vanner

**Affiliations:** 1 Gastrointestinal Diseases Research Unit, Kingston General Hospital, Queen’s University, Kingston, ON Canada; 2 Department of Dietetics, Nutrition and Sport, La Trobe University, Bundoora, VIC Australia; 3 Farncombe Institute, McMaster University, Hamilton, ON Canada; 4 Department of Gastroenterology, Monash University, Melbourne, Australia; 5 Faculty of Health Sciences, Queen’s University, Kingston, ON Canada

**Keywords:** Microbiology, Gastroenterology, Medical research

## Abstract

The lack of reproducibility of animal experimental results between laboratories, particularly in studies investigating the microbiota, has raised concern among the scientific community. Factors such as environment, stress and sex have been identified as contributors, whereas dietary composition has received less attention. This study firstly evaluated the use of commercially available rodent diets across research institutions, with 28 different diets reported by 45 survey respondents. Secondly, highly variable ingredient, FODMAP (Fermentable Oligo-, Di-, Mono-saccharides And Polyols) and gluten content was found between different commercially available rodent diets. Finally, 40 mice were randomized to four groups, each receiving a different commercially available rodent diet, and the dietary impact on cecal microbiota, short- and branched-chain fatty acid profiles was evaluated. The gut microbiota composition differed significantly between diets and sexes, with significantly different clusters in β-diversity. Total BCFA were highest (*p* = 0.01) and SCFA were lowest (*p* = 0.03) in mice fed a diet lower in FODMAPs and gluten. These results suggest that nutritional composition of commercially available rodent diets impact gut microbiota profiles and fermentation patterns, with major implications for the reproducibility of results across laboratories. However, further studies are required to elucidate the specific dietary factors driving these changes.

## Introduction

Lack of experimental reproducibility is of great concern among major funding agencies, such as the US National Institutes of Health (NIH)^1^, and the scientific community at large^2^. Of particular concern is the considerable interlaboratory variability in animal model-based microbiome studies^3^. Factors such as environment, stress and sex have been attributed to the variable findings, highlighting the importance of consistent rodent husbandry. Calls have been made to routinely characterize the gut microbiota composition to improve preclinical reproducibility and transparency^4^. Diet is increasingly recognized as a key modulator of the microbiome^5^, and has been shown to exert both local and systemic immune effects^6^.

Both human and animal studies have shown that changes in the microbiota can be induced by both short- and long-term alterations in diet, and that, among others, the carbohydrate content of the diet has a clear effect on microbiota profiles^6,7^. Dietary manipulation can be beneficial for managing functional gastrointestinal symptoms such as in irritable bowel syndrome (IBS) and inflammatory bowel disease (IBD)^8^. Dietary fermentable short-chain carbohydrates known as FODMAPs (Fermentable Oligo-, Di-, Mono-saccharides And Polyols) have gained widespread interest due to evidence of their efficacy in reducing IBS symptoms, and also for their prebiotic properties, known to impact the microbiota^8^. Likewise, dietary proteins, such as gluten, may influence gastrointestinal symptoms as well as affect gut microbiota composition^9,10^.

While much of the data for the low FODMAP and gluten-free diets come from clinical trials, which suffer from some limitations, researchers are now turning to animal models to better understand the mechanisms underlying dietary-induced changes^11,12^. A major advantage of animal studies is the ability to maintain stringent control of dietary composition. Furthermore, the ability to manipulate the microbiota, such as through the use of antibiotics and germ-free mice, provides potentially powerful tools for elucidating the mechanistic role of the microbiota^13^. The capability to strictly control experimental variables, including dietary intake and microbial composition, allows for development and rigorous testing of mechanistic hypotheses that might not be possible in human studies^13^.

Altering rodent diets results in quick but reversible changes to the gut microbiota in a range of host genotypes^14^. However, vast arrays of commercially available experimental diets with substantially diverse ingredients and nutrition profiles are currently available and used across laboratories. In light of the their marked influence on the microbiota, the heterogeneity of diets used across studies effectively limits the conclusions that one can draw^15^. Moreover, the FODMAP and gluten content of commercially available animal diets are not known.

With increased knowledge of the impact of dietary components and its influence on the microbiota, more consideration should be given to diets used in animal studies. The aims of this study were threefold: first, to evaluate what types of commercially available rodent diets are used in research and industry; second, to analyze the nutrient content, specifically the FODMAP and gluten content, of common rodent diets; and, finally, to assess the impact of different diets in vivo on microbiota profiles and fermentation patterns.

## Methods

### Use of commercially available rodent diets across institutions

On 16th May 2019, a Qualtrics survey was sent via the American Association for Laboratory Animal Science (AALAS) Listserv COMPMED, an AALAS member forum to exchange knowledge. At the time of distribution, there were 3916 Listserv recipients worldwide. The survey aimed to evaluate use of commercially available rodent diets. Respondents were asked to answer five questions regarding their institutional location, their institution type (e.g., university, industry), the type of rodent diets used at their institution, how the diets were provided to animals (e.g., given as is, irradiated prior to purchase, autoclaved on site), and their water source and its treatment (e.g., municipal tap water, autoclaved, UV sterilized). As the survey involved individuals who were not themselves the focus of the research, the survey was provided an exemption from the Queen’s University Health Sciences and Affiliated Teaching Hospitals Research Ethics Board. Informed consent was inferred when respondents opened the survey.

### Nutritional analysis of rodent diets

#### Selection and sourcing of commercially available rodent diets

Commonly available rodent diets containing a range of ingredients were selected and sourced for nutritional analysis (Table 1). As per standard protocol used for FODMAP analysis, three mill dates were collected per diet to obtain a representative sample. Diets were selected based on ensuring three different brands were included with at least two diets per brand; inclusion of both grain-based chows and purified ingredient diets; and diets that used diverse main ingredients, for example ground wheat versus ground corn as the main ingredient.

**Table 1:** Key ingredients and macronutrient composition of the commercially available rodent diets.

Rodent diet tested	Key ingredients (in order of weight)	Protein % kcal	Fat % kcal	Carbohydrate % kcal	Fibre (crude) %	kcal/g
*Purified ingredient diets*						
Research Diets, AIN93G Growing Rodent purified ingredient diet	Per 1000 g: 397.49 g Corn starch, 200 g casein, 132 g Lodex 10, 100 g sucrose, 70 g soybean oil, 50 g Solka Floc FCC200, 35 g mineral S10022G, 10 g vitamin V10037, 3 g cystine, 2.5 g choline bitartrate, 0.01 g tert-butylhydroquinone	21	12	67	5.0	3.86
Research Diets, RD Western purified ingredient diet	Per 1000 g : 350 g Sucrose, 200 g anhydrous butter, 195 g casein, 100 g Lodex 10, 50 g corn starch, 50 g Solka Floc FCC200, 17.5 g mineral S10001A, 17.5 g Calcium Phosphate Dibasic, 4 g Calcium Carbonate Light USP, 10 g corn oil, 3 g methionine, 2 g Choline Bitartrate, 1.5 g cholesterol NF, 1 g vitamin V10001C, 0.04 g ethoxyquin	17	40	43	5.0	4.67
*Grain-based chows*						
Envigo, G18% Rodent chow	Ground wheat, ground corn, wheat middlings, dehulled soybean meal, corn gluten meal, soybean oil, calcium carbonate, dicalcium phosphate, brewers yeast, iodized salt	24	18	58	3.5	3.1
Envigo, S-2335 M/R chow	Ground wheat, ground corn, dehulled soybean meal, porcine fat preserved with BHA, dried whey casein, brewers dried yeast, porcine meat and bone meal, soybean hulls, calcium carbonate, iodized salt	20	29	51	2.7	3.5
Lab Diet, 5015 Mouse chow	Whole wheat, dehulled soybean meal, ground corn, wheat germ, brewers dried yeast, porcine animal fat preserved with BHA and citric acid, condensed whey solubles, calcium carbonate, salt, dried whey protein concentrate, soybean oil, mono and diglycerides of edible fats	20	26	54	2.4	3.83
Lab Diet, 5001 Rodent chow	Ground corn, dehulled soybean meal, dried beet pulp, fish meal, ground oats, brewers dried yeast, cane molasses, dehydrated alfalfa meal, dried whey, wheat germ, porcine animal fat preserved with BHA, porcine meat meal, wheat middlings, salt	29	13	58	5.2	3.36
Lab Diet, 5021 Autoclavable Mouse chow	Ground corn, wheat middlings, dehulled soybean meal, wheat germ, fish meal, whole wheat, porcine animal fat preserved with BHA and citric acid, brewers dried yeast, soybean oil, ground oats, dried beet pulp, salt	23	24	53	3.7	3.72
Lab Diet, 5066 Rodent chow	Ground corn, wheat middlings, dehulled soybean meal, animal fat preserved with BHA, fish meal, dehydrated alfalfa meal, cane molasses, calcium carbonate, salt, ground oats, ground wheat, ground soybean hulls, dried beet pulp, wheat germ, dried whey, dl-methionine, dicalcium phosphate, menadione dimethylpyrimidnol bisulfate, corn gluten meal	21	15	65	5.0	3.4
*Custom-made diets*						
Custom-made low FODMAP purified ingredient diet (Envigo, Teklad Custom Diet TD.170455)	Per 1000 g: 349.222 g corn starch, 200 g casein, 132 g maltodextrin, 100 g sucrose, 70 g lard, 50 g cellulose, 35 g mineral mix AIN-93G-MX 94046, 30 g soybean oil, 15 g vitamin mix AIN-93G-VX 94047, 4.75 g fructooligosaccharide, 3.56 g fructose, 3 g L-Cystine, 2.8 g calcium phosphate dibasic, 2.75 g choline bitartrate, 1.2 g galactooligosaccharide, 0.6 g sorbitol, 0.1 g green food color, 0.014 g antioxidant TBHQ, 0.002 g vitamin K phylloquinone, 0.002 g biotin	18	24	58	5.6	3.9
Custom-made high FODMAP purified ingredient diet (Envigo, Teklad Custom Diet TD.170456)	Per 1000 g: 282.832 g corn starch, 200 g casein, 132 g maltodextrin, 100 g sucrose, 70 g lard, 50 g cellulose, 35 g mineral mix AIN-93G-MX 94046, 30 g soybean oil, 15 g vitamin mix AIN-93G-VX 94047, 35 g fructose, 24.0 g fructooligosaccharide, 11.5 g sorbitol, 6 g galactooligosaccharide, 3 g L-Cystine, 2.8 g calcium phosphate dibasic, 2.75 g choline bitartrate, 0.1 g green food color, 0.014 g antioxidant TBHQ, 0.002 g vitamin K phylloquinone, 0.002 g biotin	19	24	57	8.0	3.8

#### Quantification of total FODMAP content of rodent diets

Analysis of rodent diets was performed in triplicate using well-established techniques to measure FODMAP content^16,17^. Briefly, samples were ground using an electric food processor to a fine particle size (approximately 0.5 mm) prior to sugar extraction. Following sugar extraction, samples were analyzed using high-performance liquid chromatography (HPLC) employing Waters HPLC using evaporative light scattering detector (ELSD) with a Sugar Pak 1 column (6.5 9 300 mm column; Waters Corporation; New South Wales, Australia) to separate carbohydrates, then compared with standards for sucrose, glucose, fructose, mannitol and sorbitol. Analysis using ultra-performance liquid chromatography (UPLC) was completed using a Waters Acquity UPLC with ELSD detector (BEH Amide 1.7 lm column; Waters Corporation; New South Wales, Australia) to separate carbohydrates, then compared with standards for lactose, raffinose, stachyose, kestose, and nystose. Subtracting total glucose from total fructose content provided excess fructose content, whilst addition of raffinose and stachyose content provided total galacto-oligosaccharide (GOS) content. Total fructan content was determined by an enzymatic fructan assay (Megazyme Fructan HK Assay AOAC Method 999.03, AACC Method 32.32; Megazyme International Ireland Ltd; Wicklow, Ireland).

The FODMAP content of the commercial rodent diets was then compared to custom-made low- and high- FODMAP rodent purified ingredient diets that were developed to mimic that of human consumption. The custom-diets were used previously in the laboratory for separate experiments^18^. One purified ingredient diet was designed to be low in FODMAP content (Envigo Teklad Custom Diet TD.170455; Wisconsin, USA), the other designed to be high in FODMAP content (Envigo, Teklad Custom Diet TD.170456; Wisconsin, USA). The custom-made diets contained fructose (≥ 99.5% fructose, Tate and Lyle; Illinois, USA), sorbitol (100% Sorbitol powder; Spectrum Chemical MFG. CORP.; California, USA), galacto-oligosaccharide (98% Soybean Oligosaccharide powder, containing raffinose ≥ 14–18%, stachyose ≥ 55–65%, verbascose ≥ 16–20%; Hunan Nutramax Inc.; Changsha, China), and fructans (NutraFlora 95% FOS powder, containing DP3 30–42%, DP4 45–57%, DP5 5–15%; NOW Foods; Illinois, USA). Carbohydrate content, including each specific FODMAP subgroup, was matched to that of dietary studies performed in humans involving closely controlled FODMAP intake^19^.

#### Quantification of gluten content of rodent diets

Quantification was performed using a competitive G12 ELISA GlutenTox Kit (Biomedal; Seville, Spain) that recognizes gluten peptides derived of 33-mer, an immunogenic and resistant peptide to mammalian enzymes and industrial processing, according to the manufacturer’s instructions^20^.

### Impact of commercially available rodent diets on microbiota profiles and fermentation patterns

#### Animal protocol

All experiments were approved by the Queen’s University Animal Ethics Committee (Animal Protocol number 2016-1644). All protocols were conducted in accordance with the guidelines of the Canadian Council of Animal Care. Male and female cage mate C57BL/6 mice (males: 20–24 g; females: 16–20 g) were purchased Specific Pathogen Free from Charles River Laboratories (Quebec, Canada) and allowed to acclimatize to the facility for 1 week prior to commencing the protocol. During acclimatization, mice remained on the same chow that they received at the breeding institution (LabDiet 5066, derived from corn and wheat). Five animals of the same sex were housed per individually ventilated cage (Tecniplast GM500 IVC caging) containing wood chips, in a 12/12 h light/dark cycle. Mice had ad libitum access to food and water throughout the protocols. Automatic watering was used with chlorinated reverse osmosis water at 2-4 ppm. The room temperature was between 20–22 °C, and humidity between 20 and 50%.

Forty C57BL/6 mice were randomized to four groups (5 male and 5 female per group, Supplementary Fig. 1) and were weighed at the beginning and end of the protocol. Group A was euthanized at baseline and samples collected as described below. Group B received the breeding institution chow (LabDiet 5066, derived from corn and wheat). Group C received a commercially available purified ingredient diet chosen due to its lower total FODMAP and gluten content (ResearchDiets AIN93G diet, derived from corn starch), and Group D received a commercially available chow selected due to its higher total FODMAP and gluten content (LabDiet 5001; derived from corn and soybean meal). Each group received their respective diet for 3 weeks. While the rodent diets used were selected based on their FODMAP and gluten content, they also differed in quantity and sources of fat and protein content (Table 1).

**Figure 1:**
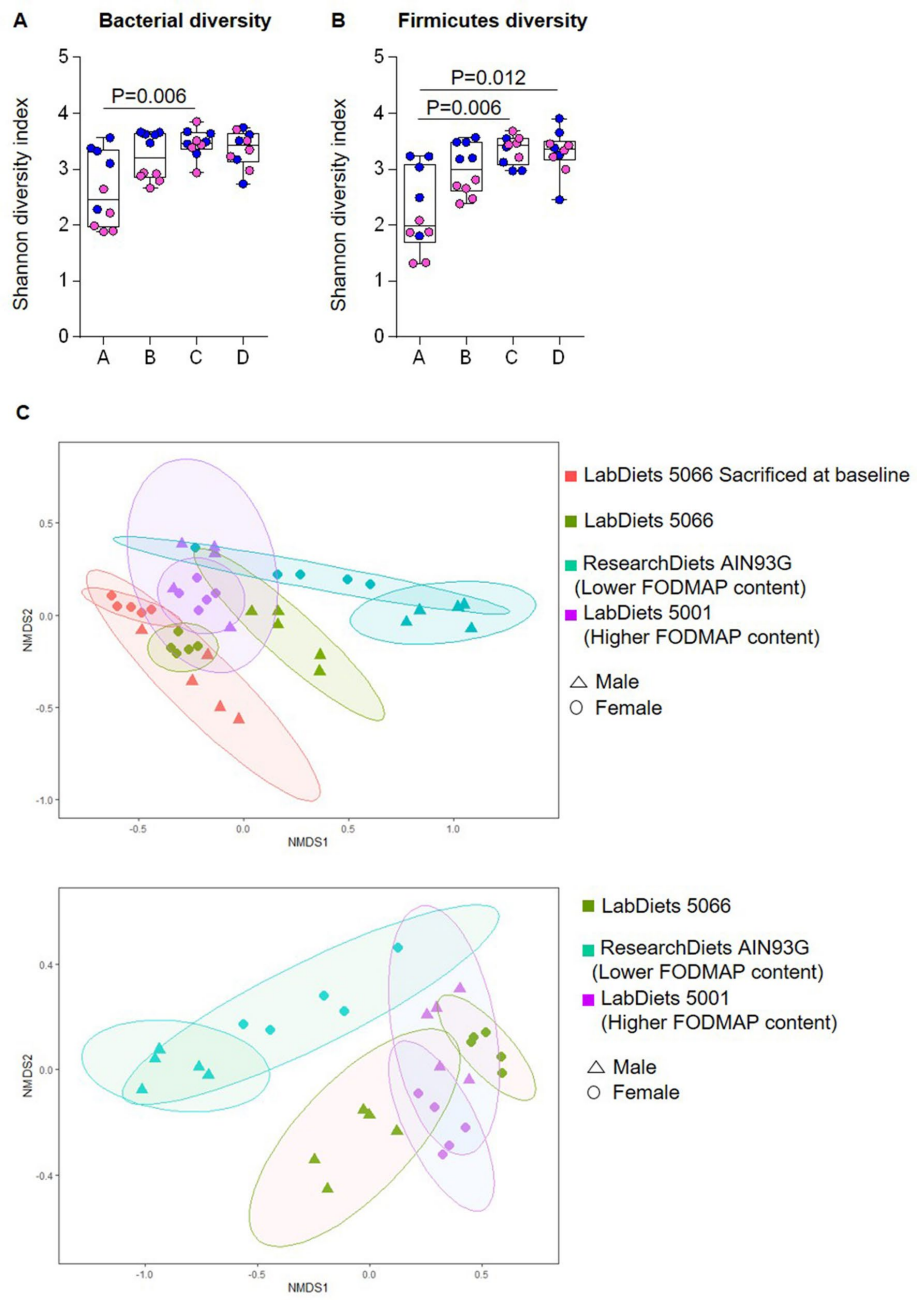
Analysis of cecal microbiota of mice receiving 4 different commercially available rodent diets. (**A**) α-diversity plot of the Shannon diversity index. Females are shown here as pink dots and males as blue dots. Sex significantly influenced microbial diversity in group A and B, both groups receiving the LabDiet 5066. The analysis was done with the script “compare_alpha_diversity.py” with the option of non-parametric testing, and *p* value was determined with Monte Carlo permutations. Multiple comparisons were corrected with Bonferroni correction. (**B**) Shannon diversity index plot of Firmicutes diversity. Firmicutes diversity was altered by diet C and D in comparison to diet A (*p* = 0.006, and *p* = 0.006, respectively). The analysis was done with the script “compare_alpha_diversity.py” with the option of non-parametric testing, and *p* value was determined with Monte Carlo permutations. Multiple comparisons were corrected with Bonferroni correction. (**C**) β-diversity plot (NMDS) constructed using the Bray–Curtis dissimilarity matrix. Cecal microbiota profiles were significantly altered by the use of different diets. Mice clustered by type of diet and sex (Adonis *p* < 0.01).

#### Sample collection

Animals were deeply sedated with isofluorane and euthanized by decapitation. The cecum and its contents, the main site of fermentation, were collected, immediately snap frozen and stored at − 80 °C until analysis.

#### 16S rRNA gene sequencing and microbiota analysis

Total genomic DNA was extracted from the cecal contents, as previously described at McMaster University’s Metagenomic Facility^21^. Following this protocol, amplification of the V3 region of the 16S rRNA gene, and Illumina sequencing was performed as previously described^22,23^. Briefly, the data were processed by an in-house bioinformatics pipeline that incorporates quality filtering, Cutadapt, PandaS, AbundantOTU, and QIIME1^23^. Abundant OTU provides as output sequences clustered in operational taxonomic units (OTUs). Taxonomic assignments was done using the RDP classifier^24^ with the Greengenes^25^ (2013) training sets.

Using 16S rRNA Illumina, a total of 2,754,012 reads (average of 68,850.3 reads, with a minimum of 22,356 and a maximum of 116,526 reads per sample), and a total of 1303 OTUs (an average of 147 OTUs per sample, with a range of 65–256) were obtained from the 40 samples analyzed. The OTU table was normalized to relative abundance. The results obtained were similar when the table was normalized with rarefaction to a common sequence depth. β-diversity was calculated using the Bray–Curtis dissimilarity matrix. All scripts used for the analyses are available upon request.

#### Short-chain fatty acid and pH measurement

Using cecal contents, quantities of SCFA (acetic, propionic, butyric, pentanoic acids) and BCFA (isobutyric, isovaleric acids) were measured as per methods previously described^26–28^. Briefly, 10 µL of internal standard (14.72 mM butyric acid-d_7_) was added to 30 mg of frozen cecal contents. After acidification with HCl, fatty acids were extracted (diethyl ether, 3–5 cycles). Samples were then incubated for 1 h with N-tert-butyldimethylsilyl-N-methyltrifluoroacetamide (MTBSTFA) and SCFA and BCFA content quantified using gas chromatography-mass spectrometry. The pH of cecal contents was measured following dilution of 1:5 with physiological saline solution on pH test papers (Hydrion- Micro Essential Laboratory, NY, USA).

### Statistical analysis

All data were analyzed using GraphPad Prism version 7.02 (GraphPad Software, CA, USA), except for the microbiota analysis. The data are presented as mean ± SEM. Normal distribution was determined by D’Agustino-Pearson omnibus normality test, Shapiro–Wilk test and Kolmogorov–Smirnov test with Dallal–Wilkinson–Lillie correction. A *p* value ≤ 0.05 was selected to reject the null hypothesis by 2-tailed tests^18^. Microbiota analyses were conducted using either QIIME1^29^, MaAsLin (Multivariate Analysis by Linear Models)^30^, Phyloseq package (1.24)^31,32^ for R (3.5), and SPSS software 23 (SPSS Inc., IL, USA). All results were corrected for multiple comparisons, allowing 5% for false discovery rate. All scripts used for the analyses are available upon request.

## Results

### Use of commercially available rodent diets across institutions

Forty-five individuals responded to the survey, 80% from universities and 20% from industry. Sixty-nine percent of respondents were from the USA, 19% from Canada, 5% from Australia, 2% from Ireland, 2% from India, and 2% from Antigua and Barbuda. Twenty-eight different types of rodent diets were reported to be used by the respondents, with the frequency used shown in Supplementary Fig. 2. The most commonly used diets were LabDiet 5001 and LabDiet 5053 each used by 14% of respondents, LabDiet 5010 was used by 10% of respondents and Envigo Teklad 19% protein and Envigo Teklad 18% protein by 7% each. Additionally, 8% of respondents used a custom formulation in their laboratories.

**Figure 2:**
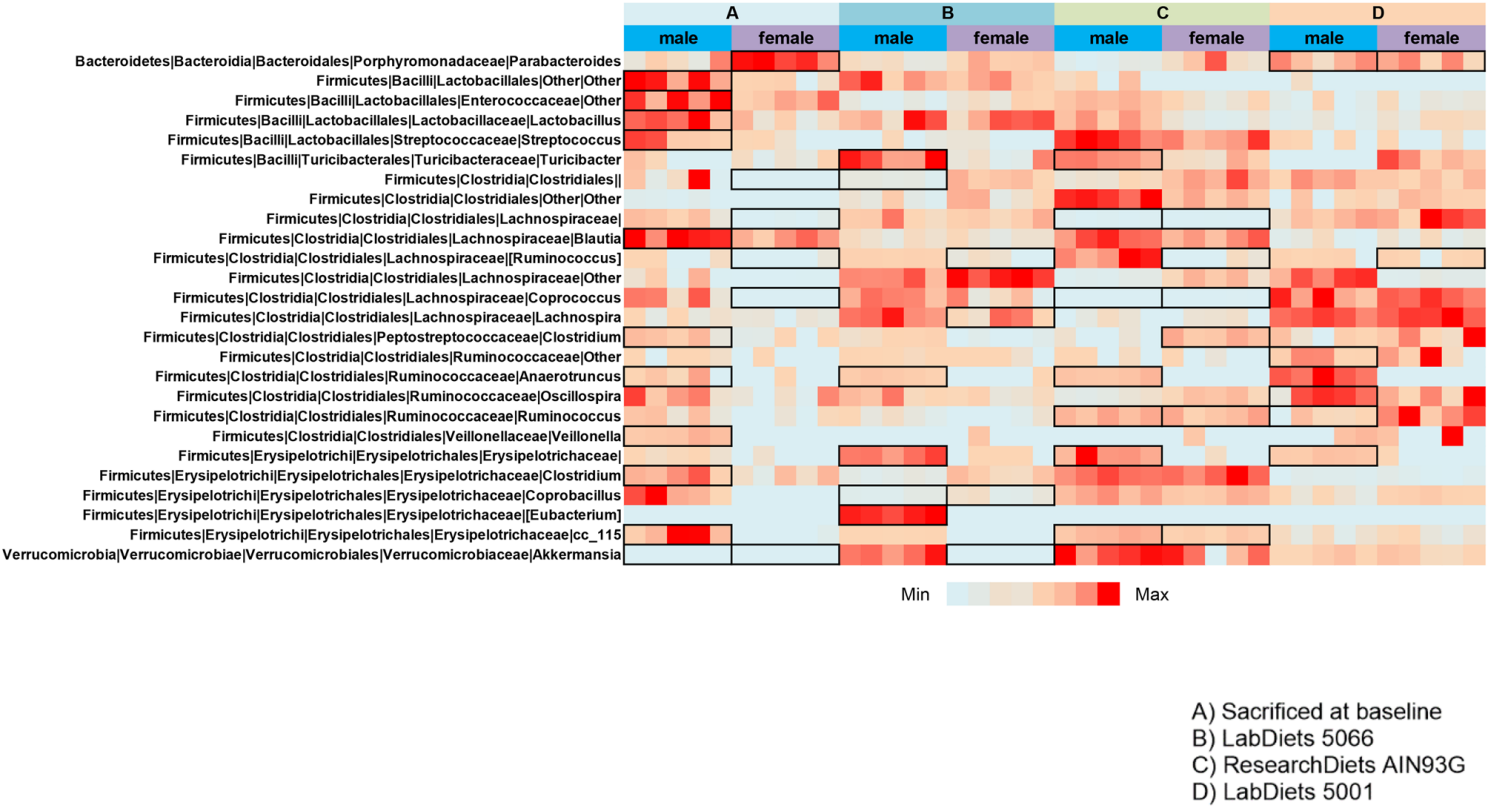
Different rodent diets significantly impact gut microbiota composition in a sex-dependent way. The data was analyzed with MaAslin to detect multivariable associations between bacterial genera and diets in each sex. Each bacterial genus significantly associated with diet and sex is highlighted by a black box.

The diets were reported to be given as purchased by 40% of respondents, purchased irradiated by 45%, autoclaved in-house by 13%, and ‘other’ by 2%. A variety of water sources were also reported, most frequently being municipal tap water (22%), reverse osmosis purified water (17%), acidified water (15%), chlorinated water (9%), autoclaved municipal tap water (8%), UV sterilized municipal tap water (8%), and ‘other’ (21%).

### Nutritional analysis of rodent diets

Seven commercially available rodent diets were selected for analysis (Table 1). Samples from three different mill dates were collected for each diet. Additionally, one sample from an autoclavable chow was also collected and analyzed. A summary of the commercially available rodent diets and key ingredients are presented in Table 1, highlighting the expected difference between ingredients of the purified ingredient diets versus the grain-base chows. Of the grain-based chows, each contained a significant portion of wheat, corn and soybean meal but in varying quantities. Of the commercially available diets, the energy content of the diets ranged from 3.1 to 4.67 kcal/g, percentage protein ranged from 17 to 29%, percentage fat from 12 to 40%, percentage carbohydrate from 43 to 67%, and crude fibre 2.4–5.0%.

#### Total FODMAP content of the rodent diets

As shown in Table 2, the total FODMAP content varied considerably among the commercially available rodent diets, with the lowest FODMAP composition found in the Research Diets AIN93G Growing Rodent purified ingredient diet derived from corn starch (total FODMAP content of 0.40 g/100 g) and the highest FODMAP content was the Lab Diets 5001 Rodent Chow derived from corn and soybean meal (total FODMAP content of 4.62 g/100 g). These diets with lowest and highest FODMAP composition were comparable in FODMAP content to the custom-made low FODMAP purified ingredients diet containing 0.51 g/100 g total FODMAP, and the custom-made high FODMAP purified ingredient diet containing 4.10 g/100 g total FODMAP.

**Table 2:** FODMAP content of the commercially available and custom-made rodent diets.

Rodent diet tested	Excess fructose (g/100 g)	Lactose (g/100 g)	Total polyols (g/100 g)	Total GOS (g/100 )g	Fructan (g/100 g)	Total FODMAP content (g/100 g)
Research diets, AIN93G Growing Rodent purified ingredient diet	0.00	0.00	0.00	0.09‡	0.31	0.40
Research Diets, RD Western purified ingredient diet	0.00	0.00	0.00	0.00‡	0.76	0.76
Envigo, G18% Rodent chow	0.03	0.00	0.00	0.63	0.85	1.51
Envigo, S-2335 M/R chow	0.00	2.59	0.01	0.71	0.61	3.93
Lab diet, 5015 Mouse chow	0.00	1.67	0.03	1.62	0.92	4.24
Lab Diet, 5001 Rodent chow	0.00	1.86	0.01	2.04	0.71	4.62
Lab Diet, 5021 Autoclavable Mouse chow†	0.05	0.00	0.00	1.14	0.90	2.09
Lab Diet, 5066 Rodent chow	0.00	0.00	0.01	1.02	0.83	1.86
Custom-made low FODMAP purified ingredient diet†	0.07	0.00	0.00	0.00	0.44	0.51
Custom-made high FODMAP purified ingredient diet†	1.70	0.00	1.14	0.26	1.65	4.1

All samples analyzed as n = 3 except those marked with † denoting analysis as n = 1. Excess fructose is calculated by subtracting glucose content from fructose content. Total polyols is calculated as the sum of sorbitol and mannitol. Total GOS is calculated as the sum of raffinose and stachyose except where indicated ‡, as these samples contained maltodextrin or other ingredients which interferes with the analysis, hence total GOS was analyzed via a GOS enzyme kit. FODMAP denotes fermentable oligosaccharides, disaccharides, monosaccharides and polyols. GOS denotes galacto-oligosaccharides.

The variability of the total FODMAP content between commercially available diets reflected a significant variability within the FODMAP subgroups (Table 2). For example, three of the diets contained lactose (1.67–2.59 g/100 g) whilst the others contained no lactose. In regard to total polyol content, three diets contained polyols, of which only sorbitol was present (0.01–0.03 g/100 g). None of the diets contained mannitol. Total GOS content included a mixture of raffinose (0.13–0.61 g/100 g) and stachyose (0.36–1.59 g/100 g). Fructan content was also variable, ranging from 0.31–0.90 g/100 g. The total oligosaccharide content, the FODMAP subgroup most likely to alter the microbiome^33^, varied from 0.40–2.75 g/100 g (calculated as the addition of total GOS and fructan content).

#### Gluten content of the rodent diets

The gluten content of the rodent diets is displayed in Table 3. The gluten content was also highly variable between the commercially available rodent diets, but primarily reflected the main ingredients present in each diet as expected (Table 1). The lowest gluten content of 0.17 µg/mg was seen with the Research Diets AIN93G purified ingredient diet, derived from cornstarch, and the highest of 7.25 µg/mg in the Lab Diet 5066 chow, derived from ground corn and wheat middlings.

**Table 3:** Gluten content of commercially available and custom-made rodent diets.

Rodent diet tested	Gluten determination (µg/mg)
Research diets, AIN93G growing rodent purified ingredient diet†	0.17
Envigo, G18% rodent chow†	6.62
Envigo, S-2335 M/R chow†	4.97
Lab Diet, 5015 mouse chow†	6.58
Lab Diet, 5001 rodent chow	3.80
Lab Diet, 5066 rodent chow	7.25
Custom-made low FODMAP purified ingredient diet†	0.46
Custom-made high FODMAP purified ingredient diet†	0.26

All samples analyzed as n = 3, except those marked with † denoting analysis as n = 1.

### Impact of commercially available rodent diets on microbiota profiles and fermentation patterns

Forty C57BL/6 mice were randomized to four groups. Group A were euthanized at baseline, Group B received the breeding institution chow (LabDiet 5066), Group C received ResearchDiets AIN93G purified ingredient diet chosen due to its lower total FODMAP and gluten content, and Group D received LabDiet 5001 chow chosen due to its higher total FODMAP and gluten content.

There was no difference in the weights of the mice between groups at the beginning of the protocol. In the female mice, there was a statistical difference between Group B (mean ± SEM 20.68 ± 0.41 g) and Group D (18.11 ± 0.34 g) at the end of the protocol (*p* < 0.01, one-way ANOVA), no other between group differences occurred. Compared to their respective weights at baseline in the female mice, Groups B and C had a significant increase in weight at the end of the protocol (Group B 17.42 ± 0.47 g vs. 20.68 ± 0.41 g, *p* < 0.01; Group C 17.02 ± 0.24 g vs. 19.11 ± 0.47 g, *p* = 0.02), but Group D did not reach significance (17.88 g ± 0.23 vs. 18.11 ± 0.34 g, *p* > 0.99). In the male mice, Group C had a significant increase in weight from the beginning to the end of the protocol (21.03 g ± 0.39 vs. 24.28 ± 1.22 g, *p* = 0.01), while in Groups B and D the increase in weight did not reach significance (Group B 23.27 ± 0.76 g vs. 25.18 ± 2.0 g, *p* = 0.59; Group D 23.43 ± 0.24 g vs. 24.66 ± 0.46 g, *p* > 0.99).

#### Effect of commercially available rodent diets on microbiota composition

Microbiota analysis of cecal contents revealed that different diets altered gut microbial composition, as reflected by significant changes in α-diversity and microbiota clusters in β-diversity analysis (Fig. 1). Specifically, diet C significantly increased the total bacterial diversity (Shannon diversity index) in comparison to diet A (*p* = 0.006). At the Phylum level, Firmicutes diversity was higher in mice fed diet C and D in comparison to diet A (*p* = 0.006 and *p* = 0.01, respectively, Fig. 1). Statistically significant lower relative abundance of Proteobacteria and higher Verrucomicrobia were observed in Groups B, C, and D in comparison to Group A, while lower relative abundance of Bacteroidetes was seen in Groups B and C. Mice fed diets with different FODMAP content had different profiles of cecal bacteria (Supplementary Fig. 3).

**Figure 3:**
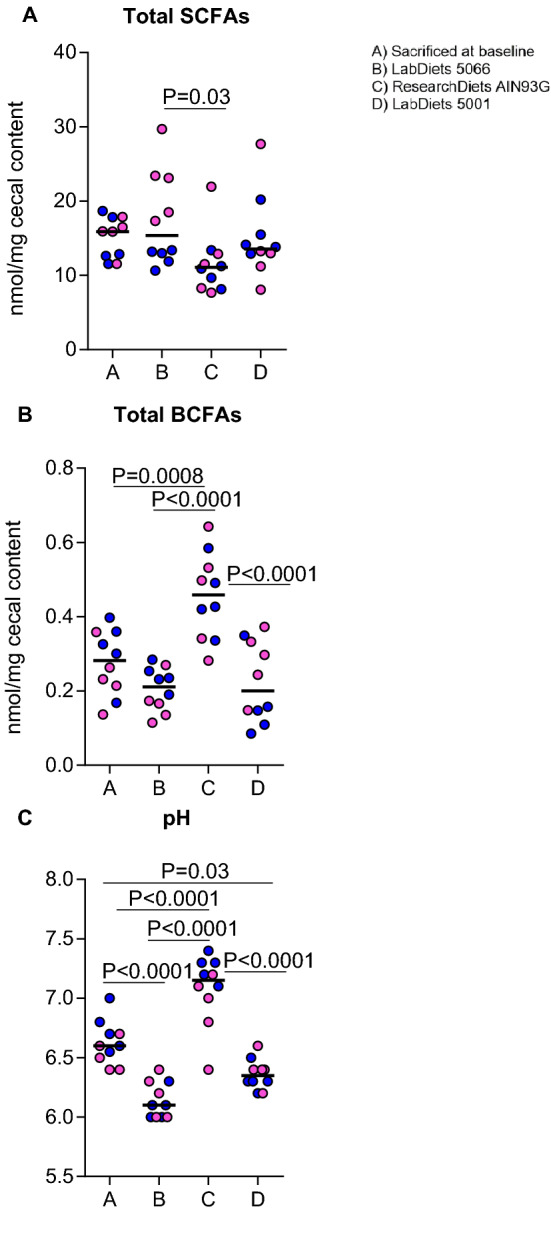
Analysis of cecal microbiota of mice receiving 4 different commercially available rodent diets. (**A**) Short-chain fatty acid (SCFA) content, (**B**) Branched-chain fatty acid content (BCFA), and (**C**) luminal pH. Females are shown here as pink dots and males as blue dots. Statistical analysis were conducted via one-way ANOVA or Kruskal Wallis test, as appropriate, and *p* values were corrected with Dunn's multiple comparisons test or Tukey’s multiple comparison test, as appropriate. The Statistics were: χ^2^(24) = 8.974, *p* = 0.029 for A, F(3,36) = 14.49, *p* < 0.0001 for B, and F(3,36) = 41.17, *p* < 0.0001 for C.

In addition, the different diets used in this study also impacted gut microbiota in a sex-dependent way. We observed sex differences for bacterial diversity (Shannon diversity index) in Groups A and B (*p* < 0.01), both groups receiving the rodent chow LabDiet 5066 used by the breeding institution (Fig. 1). Taxonomic composition was also changed in a sex-dependent way by the different diets (Fig. 2).

#### Effect of commercially available rodent diets on short- and branched-chain fatty acid composition

SCFA and BCFA were quantified from cecal contents in each group (Fig. 3). Total SCFA levels were lower (*p* = 0.03) and BCFA were higher (*p* = 0.01) in Group C, which received cornstarch-derived purified ingredient diet, compared with those of Group B receiving the chow derived from corn and wheat. The BCFA concentrations were also higher in Group C compared to Group D receiving the chow derived from corn and soybean meal. Differences in SCFA content were primarily driven by acetic acid and valeric acid, while butyric acid and propionic acid concentrations remained largely unchanged between the four groups (Supplementary Fig. 4). Both iso-valeric and iso-butyric acid levels were higher in Group C, driving the change in total BCFA content (Supplementary Fig. 4). The pH of the cecal contents was statistically higher in Group C, fed the purified ingredient diet derived from cornstarch, compared to all other groups (*p* < 0.01, Fig. 3). There was also a significant difference between the pH of Groups A and B, both groups receiving the LabDiet 5066 chow but with sample collection taken 3 weeks apart.

## Discussion

Preclinical research is fraught with reproducibility issues often limiting the generation of interstudy or interlaboratory conclusions. The current study highlights that diet may underlie poor reproducibility, particularly in those relating to the gut microbiota, by evaluating aspects of commercially available rodent diets that are readily available and widely used among the scientific community. Commercial rodent diets differ in their nutrient composition and such variation appears to have a direct effect on the gut microbial community structure and on functional outputs, such as pH and SCFA profiles. These findings have major implications for the interpretation and reproducibility of study results.

Calls have been made for the details of animal diet composition to be part of the key criteria that authors should include in reporting research studies^34–36^. Specific guidelines, such as the ARRIVE (Animal Research: Reporting of In Vivo Experiments) guidelines, have been developed to improve reporting methods^37^. Alarmingly, many researchers using rodent models are unaware of the content of the diet used and how significantly it can vary between studies^5^. In addition, the exact formulas of commercial chows are unavailable as they are proprietary^36^. The current study supports how integral the dietary composition information is for interpretation of preclinical studies. First, a broad range of commercially available rodent diets are used in research institutions, as indicated by our survey, and differences exist in how the diets are subsequently prepared and provided; for example they may be given as purchased, purchased irradiated, or autoclaved in-house. Additionally, the treatment of the water source varies across research institutions which has previously been shown to alter the microbiota resulting in differences in disease progression^38^. Second, large variations between commercially available rodent diets were noted in the ingredient composition, percentage and profile of macronutrients, content and type of fibre and FODMAPs, and gluten content, all factors which are of major relevance to the gut microbiota. The most significant compositional variation is between the purified ingredient diets (made with refined ingredients) compared to grain-based chows (made with agricultural by-products such as ground wheat, corn and soybean meals), but significant differences also exist among the purified ingredient diets and among the grain-based chows. Previous studies have addressed the influence of diet on experimental outcomes. One study in mice compared three diets—a commercially available rodent grain-based chow, a purified diet and a custom-designed diet—and showed that only the purified diet had protective effects against gut inflammation induced by dextran sulphate sodium (DSS), and that the effect was dampened when the microbiota was disrupted through antibiotics or germ-free conditions^6^. In an evaluation of the effect of 32 unique diets, differing in macronutrient composition and fibre source, on susceptibility to DSS-induced colitis and intestinal permeability in mice, fecal microbiota was variable with alterations in diet, protein exacerbated colitis via a microbiota-dependent mechanism, and certain fibres alleviated colitis of which some of the effect was microbiota-dependent^39^. The current identification of considerable differences in protein and fibre composition across diets has major implications for the effect of commercially available diets on the outcome of research studies. Grain-based chows are also known to contain biologically relevant quantities of non-nutrients and contaminants such as phytoestrogens, arsenic, heavy metals, mycotoxins, endotoxins, pesticides and pollutants which have also been shown to affect rodent phenotype, compared to purified ingredient diets which are free of such contaminants^36^. Further understanding is needed of the influence of diet, be it purified ingredient diets or grain-based chows, on the microbiota and subsequent disease susceptibility and progression.

This is the first study to assess the FODMAP composition of commercially available rodent diets, a potential factor contributing to altered microbiota profiles^8^. The content of FODMAP subgroups with well-described prebiotic actions that are most likely to alter the structure of gut microbiota, fructans and galacto-oligosaccharides, were variable between commercially available rodent diets. Gluten is another food component known to influence gut microbial profiles in mice^40^ and humans^41^, and its content generally reflected that of FODMAPs. Gluten and fructans co-exist in grains such as wheat^42^; hence it is unsurprising that diets tended to be either high or low in both gluten and FODMAP composition. It is important for researchers to realize that neither high nor low FODMAP^19^ or gluten^43^ content is necessarily unfavourable, but that they should be aware of the content of the diets and, accordingly, select a diet based on its likely influence on study design. An additional consideration for researchers when selecting rodent diets is the type of fibre used within the diet formulation. Grain-based chows such as the Lab Diet 5015 chow contain fibres from ingredients such as whole wheat and wheat germ, which will contain FODMAPs and gluten. Whereas diets such as the purified ingredient AIN93G diet will have fibre added in the form of cellulose, a longer-chain polysaccharide. These differing fibre forms will have variable effects on transit time and microbiota composition and should be considered during study design^36,44^. Many other factors of the dietary composition have the potential to alter the microbiota and other functional outputs. For example, particle size of wheat bran contained within a mouse chow was noted to influence the hepatic and systemic inflammatory markers, in particular a smaller particle size of wheat bran (150 µm) had anti-inflammatory effects and reduced caecal *Enterobacteriaceae* compared to larger particle size (1690 µm)^45^. The additional differences between purified ingredient diets and grain-based diets found in this study, including FODMAP and gluten content, support previous data suggesting results from studies using these different types of diets should not be compared^44^. These variations again highlight that dietary composition needs to be clearly stated in all animal research publications.

One major advantage of using animal models in microbiota studies is the ability to collect samples more proximally in the gastrointestinal tract. In contrast, human studies often assess the microbiota and SCFA content of stool samples due to ease of access, but this may not reflect what occurs in the proximal colon. The current study determined the microbiota profile and SCFA content of the caecal contents, as this is the major site of fermentation for mice^13^. One study assessing the microbiota in different regions of the mouse gut in response to husbandry-associated factors found that the more prominent changes in microbiota profiles occurred in the cecum compared to other regions^46^. The authors found that rodent housing and bedding both influence the cecal microbiota profile more so than the jejunal, ileal or fecal microbiota profiles. The findings of the current study also suggest that the caecal microbiota profile is influenced by dietary composition. Interestingly, the microbiota profile of the two groups of mice (Groups A and B) that remained on the Lab Diet 5066 chow had the strongest differences based on sex compared to the other diets. However, when analysing SCFAs, we observed sex differences between Group A and Group B. Group A was euthanized 1 week after acclimatization to the facility, while Group B was euthanized 3 weeks later. Although it is generally accepted that 1 week is sufficient for acclimatization after transport to a new facility, it is possible that it may take in some cases 2–3 weeks to fully acclimatize which may explain the disparity between these two groups on the same diet^47^. Additional reasons for the disparity may include age differences and exposure to alternate husbandry staff.

As the mice on each diet experienced the same housing and dietary conditions, the observations of sex differences in microbiota profiles suggests subtle sex-dependent differences in the microbiome-diet interaction and/or in the interplay between the microbiome and host gastrointestinal system and nutrient use. The microbiota has also been shown to be influenced by sex, with suggestion that longer transit time in male animals may contribute to observed differences, due to larger size allowing those bacteria that prefer to live in close contact to the epithelium time to adhere^48^. Other studies have also demonstrated sex-dependent effects of diet on the gut microbiota^49^, including increased bacterial richness in female rats while decreased in males following oligofructose supplementation^50^. Differences in SCFA production between sexes have also been demonstrated in response to oligofructose supplementation, with higher butyrate production seen in male than female mice, possibly as a result of differences in available fixed-nitrogen used to support the microbiota^50^. Weight gain and body fat composition differed between male and female mice in response to a high-fat high-sucrose diet, suggesting sex-dependent differences may occur in response to nutrients^51^. Genetic differences between sexes including hormone production may be additional factors^48,52^. Not taking sex into account has been another key criticism of rodent literature and cited as a contributor to poor reproducibility. Hence, researchers are urged to conduct trials using animals of both sexes^34,53^.

Total SCFA content was lower in Group C receiving the purified ingredient diet derived from cornstarch compared with Group B receiving the chow derived from corn and wheat. While the exact ingredients leading to changes in SCFA cannot be identified from this study, previous studies have noted that both gluten^54^ and FODMAPs^55^ can impact SCFA composition. Examination of the individual SCFAs showed that Group C had reduced acetic acid and elevated valeric acid concentrations, compared to the other three groups. Valeric acid is a potent inhibitor of histone deacetylase (HDAC)^56^ and has been associated with anti-carcinogenic and anti-inflammatory effects^57,58^. Controversy exists over the role of acetic acid, with some studies indicating a therapeutic role in lowering cholesterol and triglyceride production^59^, whereas others have reported a negative correlation to de novo cholesterogenesis and lipogenesis^60^. BCFA content was also impacted, more specifically, iso-valeric acid and iso-butyric acid levels were highest in Group C, which correlates with previous findings in mice given a low FODMAP diet compared to those given a high FODMAP diet^18^. The production of BCFAs primarily arises from microbial fermentation of undigestible protein. Thus, the elevated levels of BCFAs may reflect, in part, a switch to greater protein metabolism in light of the diets’ carbohydrate sources that provide lower carbohydrate content available for colonic fermentation. BCFAs have been reported to impart physiological benefits, including reducing circulating levels of glucose and insulin, upregulated synthesis of the satiety hormone pancreatic peptide, YY, and decreased inflammation in adipose tissues^61^. On the contrary, a recent study has linked isobutyrate to hypercholesterolemia^62^, and higher proteolytic fermentation, which is generally thought to be detrimental to the colonic microenvironment due to other end-products of protein fermentation such as hydrogen sulphide and ammonia^10^. Additionally, the higher pH in Group C compared to the other groups indicates lower rates of carbohydrate fermentation, which correlates with previous findings that a low FODMAP diet increases pH^18^, and that fructan significantly decreases pH indicating increased fermentation^63^. Overall, the differences seen in SCFA, BCFA, and pH have implications for the reproducibility of rodent studies.

Several strengths and limitations should be considered in the interpretation of the current results. The response rate to the survey was relatively low (1.1%) and may not have been representative. This might be due to the nature of the forum in which the survey was shared on, the survey may not have been applicable to all members of the forum, hence this could have impacted the response rate. However, despite the small sample, the survey was able to show a large variation in use of commercial diets across institutions that responded, highlighting that the type of diet used needs consideration in designing and interpretation of study results from different institutions. The use of commercially available diets in this study is a major strength, as it provided valuable insights on how diets might affect gut microbiota composition and therefore influence reproducibility. As a focus in this study, FODMAP and gluten content were measured, and while these were different between diets, no conclusions can be drawn as to which component(s) of the commercially available diets had key influence on the observed differences in microbiota and fermentation patterns. Based on our previous studies using the custom low-FODMAP and custom high-FODMAP purified ingredient diets, *streptococcaceae* was lower in control mice fed the high-FODMAP diet compared to control mice fed the low-FODMAP diet^18^, suggesting that the changes to the microbiota seen in the current study may have been in part driven by the differences in FODMAP content. However, due to the variation in many aspects of the diets such as macronutrient sources and percentage contribution, the results cannot elucidate the specific dietary compositional factors driving change. The collection of three mill dates per diet for the FODMAP analysis is an additional strength of the study, especially considering that the nutritional content varies from batch to batch depending on agronomical market fluctuations^13^. Within the current study, four of the rodent diets were tested for FODMAP content individually, with data pooled after analysis. Overall, limited variation was seen in FODMAP content between mill dates (data not shown). Importantly, in order to assess sex differences both male and female mice were included in the protocol. It is known that caging and bedding can influence the microbiota^46^; consequently, mice were housed in groups of five to reduce stress and avoid potential differences in microbiota composition due to single-housing of mice. Considering our survey identified a range of diet treatments are used across institutions (i.e. given as is, purchased irradiated, autoclaved in-house), future studies may consider assessing the effect of diet treatment on microbiota profiles and subsequent disease response.

In conclusion, this study highlights a considerable variation in the choice of commercially available rodent diets across research institutions, as well as the wide variation in nutrient composition of rodent diets. Secondly, these findings provide clear evidence that the dietary composition of commercially available diets directly influences microbiota composition, leading to altered fermentation patterns, although the specific components of the diets driving the change could not be elucidated. In addition, sex is another strong factor influencing the microbiome profile. Overall, these results have major implications for the reproducibility of results across laboratories and highlight the need for all research publications to provide detailed information on the composition of diets used.

## Supplementary information

Supplementary Information 1
