# Supplementary material for: Nutritional profile of rodent diets impacts experimental reproducibility in microbiome preclinical research
Source: Sci Rep. 2020 Oct 20;10:17784. doi: 10.1038/s41598-020-74460-8 (PMC7575541; doi:10.1038/s41598-020-74460-8)

Title: Nutritional profile of rodent diets impacts experimental reproducibility in microbiome preclinical research

Authors: CJ Tuck[1,2+*], G De Palma[3+], K Takami[1], B Brant[1], A Caminero[3], DE Reed[1], JG Muir[4], PR Gibson[4], A Winterborn[5], EF Verdu[3], P Bercik[3], S Vanner[1]

SUPPLEMENTARY FIGURES

Supplementary Figure 1. ***In vivo*** **study protocol**

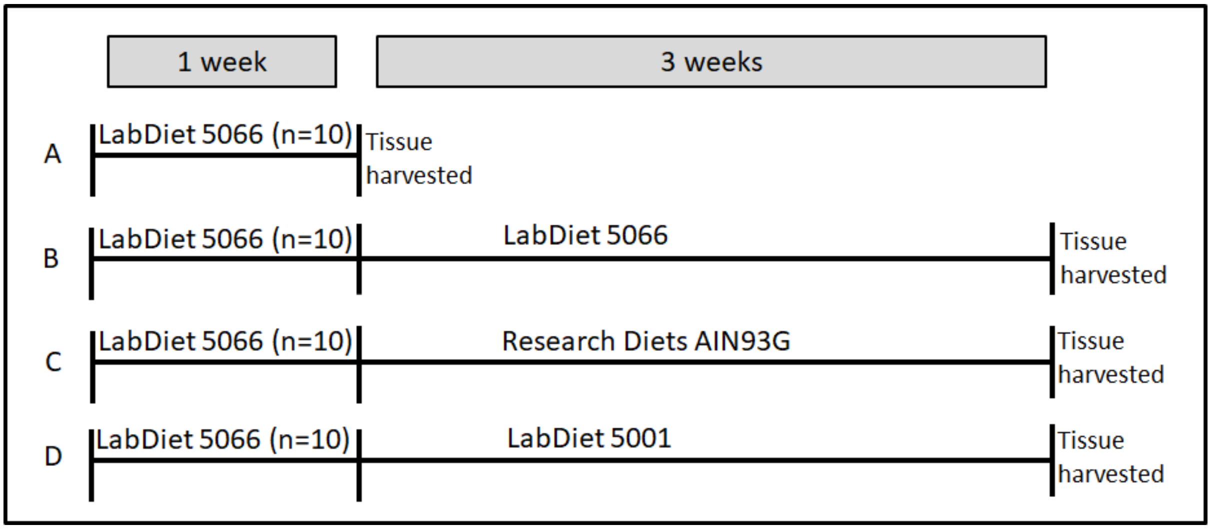

Supplementary Figure 2. Diets used in the United States of America, Canada, Australia, India, Ireland, Antigua and Barbuda. A wide variety of commercially available rodent diets were reported to be used by universities and industry. Those marked in black indicate diets assessed in dietary compositional studies (study aim 2).

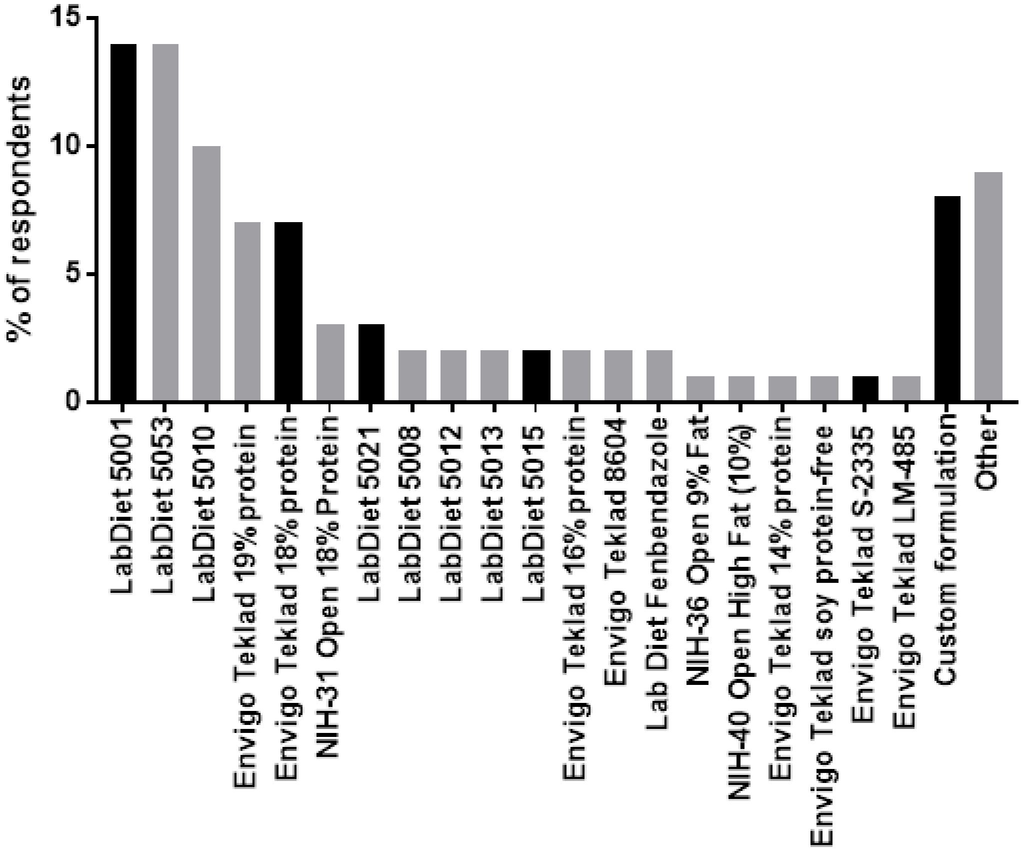

Supplementary Figure 3. The impact of different commercially available rodent diets on gut microbiota composition. Analysis conducted using MaAsLin (Multivariate Analysis by Linear Models)[26] of the taxonomy table at genus level. All P values were corrected for FDR, allowing a 5% of FDR.

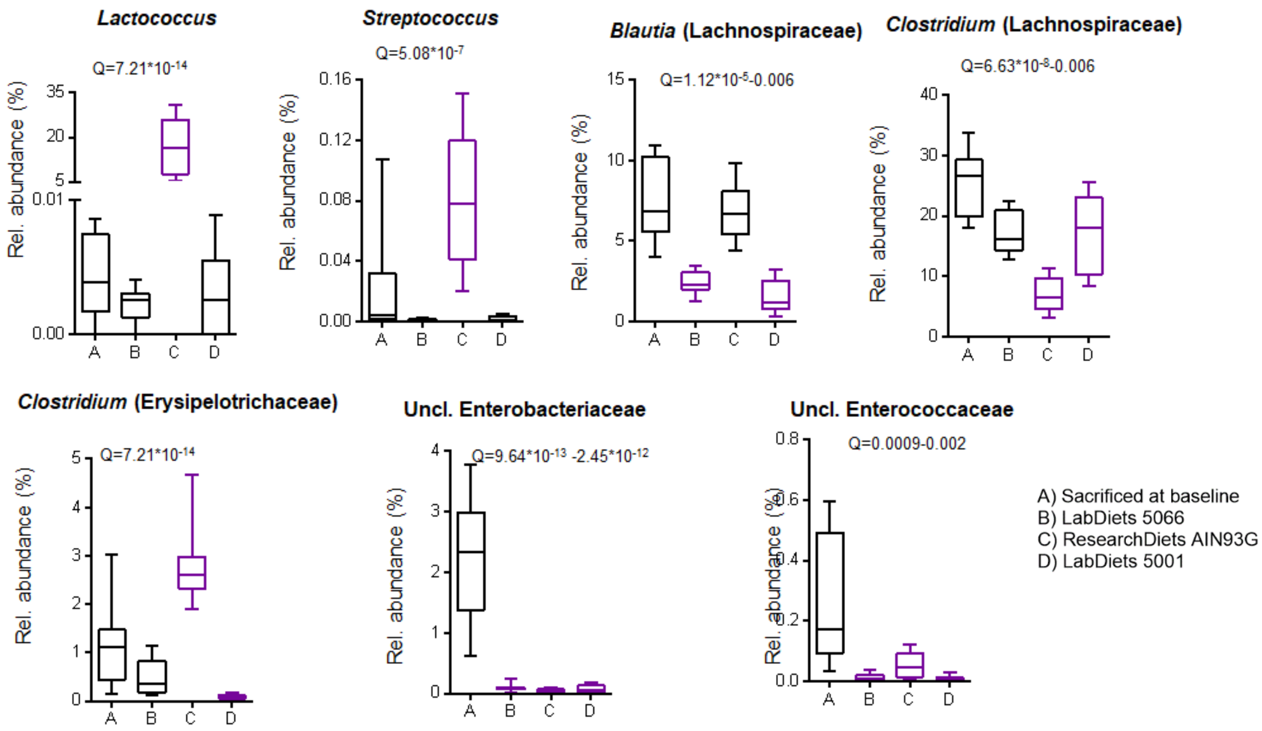

In violet the diets that were significantly associated with changes in the respective bacterial genera

Supplementary Figure 4. (A) Short-chain fatty acid analysis of cecal contents (displayed with median). (B) Branched-chain fatty acid analysis of cecal contents (displayed with mean). Females are shown here as pink dots and males as blue dots. Statistical analysis conducted via one-way ANOVA.

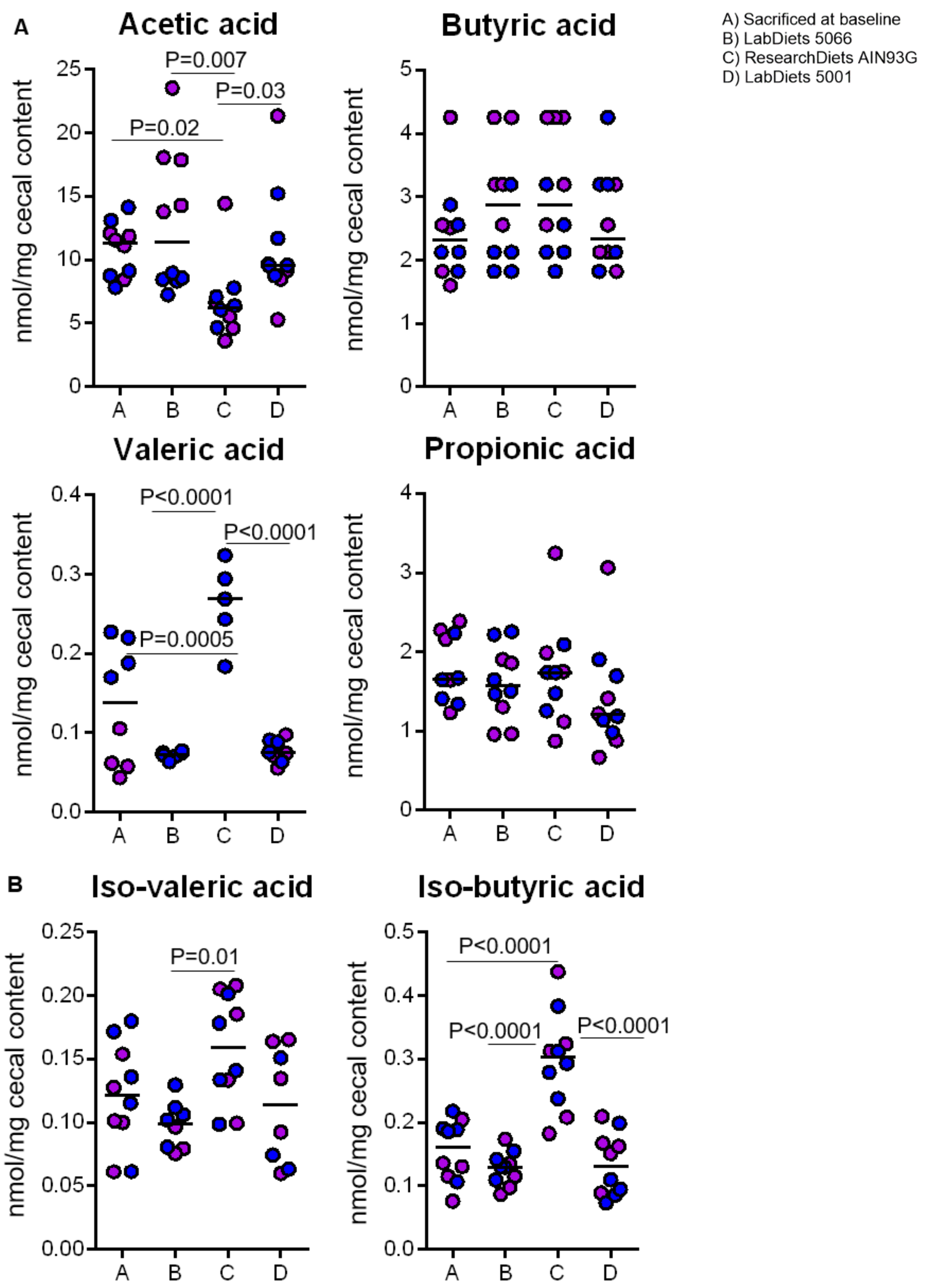

Supplement: Supplementary file 1 — Supplementary Information 1 [file 41598_2020_74460_MOESM1_ESM.pdf]
